# New polyp image classification technique using transfer learning of network-in-network structure in endoscopic images

**DOI:** 10.1038/s41598-021-83199-9

**Published:** 2021-02-11

**Authors:** Young Jae Kim, Jang Pyo Bae, Jun-Won Chung, Dong Kyun Park, Kwang Gi Kim, Yoon Jae Kim

**Affiliations:** 1 Department of Biomedical Engineering, Gil Medical Center, Gachon University College of Medicine, 21, Namdong-daero 774 beon-gil, Namdong-gu, Incheon, 21565 Republic of Korea; 2 Division of Gastroenterology, Department of Internal Medicine, Gachon University Gil Medical Center, 21 Namdongdaero 774 beon-gil, Namdong-gu, Incheon, 21565 Republic of Korea

**Keywords:** Gastroenterology, Biomedical engineering

## Abstract

While colorectal cancer is known to occur in the gastrointestinal tract. It is the third most common form of cancer of 27 major types of cancer in South Korea and worldwide. Colorectal polyps are known to increase the potential of developing colorectal cancer. Detected polyps need to be resected to reduce the risk of developing cancer. This research improved the performance of polyp classification through the fine-tuning of Network-in-Network (NIN) after applying a pre-trained model of the ImageNet database. Random shuffling is performed 20 times on 1000 colonoscopy images. Each set of data are divided into 800 images of training data and 200 images of test data. An accuracy evaluation is performed on 200 images of test data in 20 experiments. Three compared methods were constructed from AlexNet by transferring the weights trained by three different state-of-the-art databases. A normal AlexNet based method without transfer learning was also compared. The accuracy of the proposed method was higher in statistical significance than the accuracy of four other state-of-the-art methods, and showed an 18.9% improvement over the normal AlexNet based method. The area under the curve was approximately 0.930 ± 0.020, and the recall rate was 0.929 ± 0.029. An automatic algorithm can assist endoscopists in identifying polyps that are adenomatous by considering a high recall rate and accuracy. This system can enable the timely resection of polyps at an early stage.

## Introduction

Gastrointestinal (GI) disease is one of the most common diseases. Colorectal cancer arises in the GI tract. This is the third most common cancer of 27 major cancers in South Korea and worldwide^1,2^. Polyps are important since they can cause colon cancer. According to adenoma-carcinoma sequence theory, 95% of sporadic colon cancers are caused by polyps. A colon polyp is cell crowding that can develop on the mucosa of the colon, and appears in various shapes^3^.

The cumulative risk of cancer developing in an unremoved polyp is 2.5% at 5 years, 8% at 10 years, and 24% at 20 years after diagnosis^4^. Endoscopy is widely used for the diagnosis and treatment of gastrointestinal diseases. Colonoscopy is important in the prevention of colon cancer through polypectomy. If detected at an early stage, these polyps can be easily removed. Therefore, it is very important to detect all polyps. However, colonoscopy is a time-consuming and repetitive task. Sometimes, the endoscopist may have tired eyes and suffer from an attention deficit. According to the experience and evaluation standards of doctors, the failure rate for polyp detection varies from 22 to 28%^5–8^.

Recently published papers dealt with polyp classification based on deep learning methods in endoscopic images^9–12^. Zhang et al. proposed a classification method that transferred low-level convolutional neural network (CNN) features from the non-medical domain^10^. This method extracted features from convolutional layers and used these features in a support vector machine classifier. This research used the 2012 version of ImageNet challenge and Places205 for pretraining models, and produced reasonable accuracy. Billah et al. proposed a combination method of color wavelet features and convolutional neural network features, and applied a support vector machine classifier with combined features^9^. Tajbakhsh et al. made a polyp classification method based on a unique three-way image presentation and convolutional neural networks. This method learned a variety of polyp features such as color, texture, shape, and temporal information in multiple scales. Park et al. proposed a method to learn hierarchical features using a convolutional neural network. The features were learned in different scales to provide scale-invariant features through the convolutional neural network.

It has become increasingly common within the computer vision community to treat image classification on already trained pre-trained models for training deep convolutional neural networks in order to learn good general-purpose features^13,14^. The proposed method uses a pre-trained model in NIN^15^ for polyp classification and compares the performance with several pre-trained models in AlexNet^16^. The used pre-trained models are Places205^17^, ImageNet (ILSVRC12 version)^16^, and the Salient Object Subitizing (SOS) dataset^18^. Despite the importance of benchmarks and training datasets in computer vision, comparing datasets is still an open problem. Even datasets covering the same visual classes have notable differences that result in different generalization performances when they are used to train a classifier^19^. This paper analyzed the system performance according to a sort of pre-trained model databases, and proposed the best structure for a convolution neural network to classify polyps.

## Materials and methods

### Dataset

**Figure 1:**
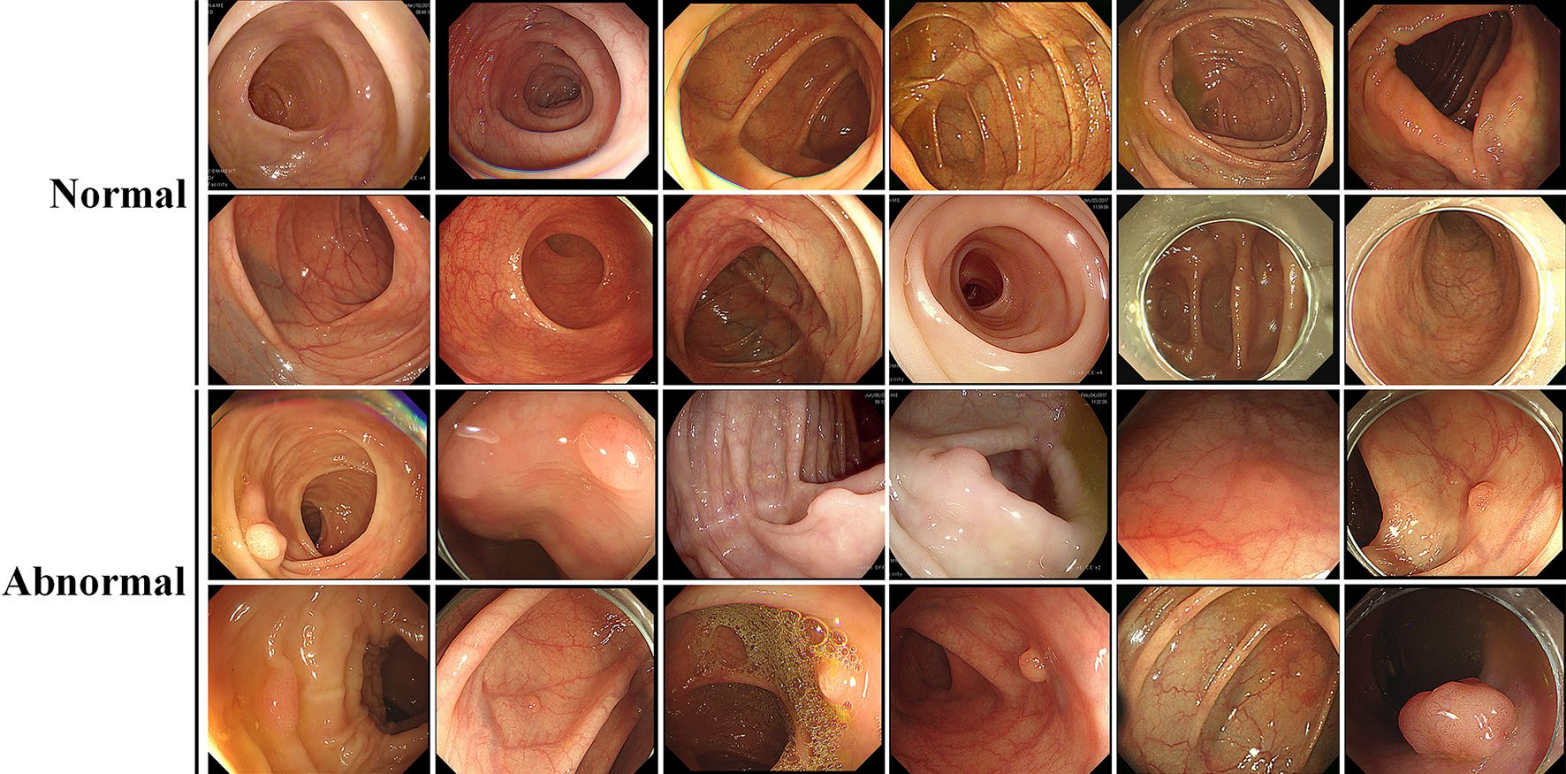
Samples of colonocopy images used in the experiment.

The Institutional Review Board of Gachon University Gil hospital approved (IRB No. GAIRB2018-051) this retrospective study and waived the requirement for informed consent for both study populations. All methods were performed in accordance with the relevant guidelines and regulations. The number of experimental data is 1000, and images are captured during colonoscopy. While counts of normal data are 500, the number of images containing polyps is 500 (Fig. 1). The original size of an image is 2072 by 1776 (width by height), and this image is resized as 227 by 227 in (width by height). The channel size of the image is 3 because the original images have RGB color. All data were acquired from the children’s medicine department of Gachon University Gil hospital in South Korea.

Random shuffling is performed 20 times on these 1000 data. Each set of data is divided into 640 images of training data (normal: 320 images, polyp: 320 images), 160 images of validation data (normal: 80 images, polyp: 80 images) and 200 images of test data (normal: 100 images, polyp: 100 images). During the training, a pre-trained model can be used, and an accuracy evaluation is performed on 200 images of test data in 20 experiments. This database is trained using the Caffe package on a GPU Nvidia Titan X. All models were trained by the same hyperparameters under the conditions of 1000 epochs, 64 batch size, and 0.0001 learning rate.

### AlexNet based transfer learning

**Figure 2:**
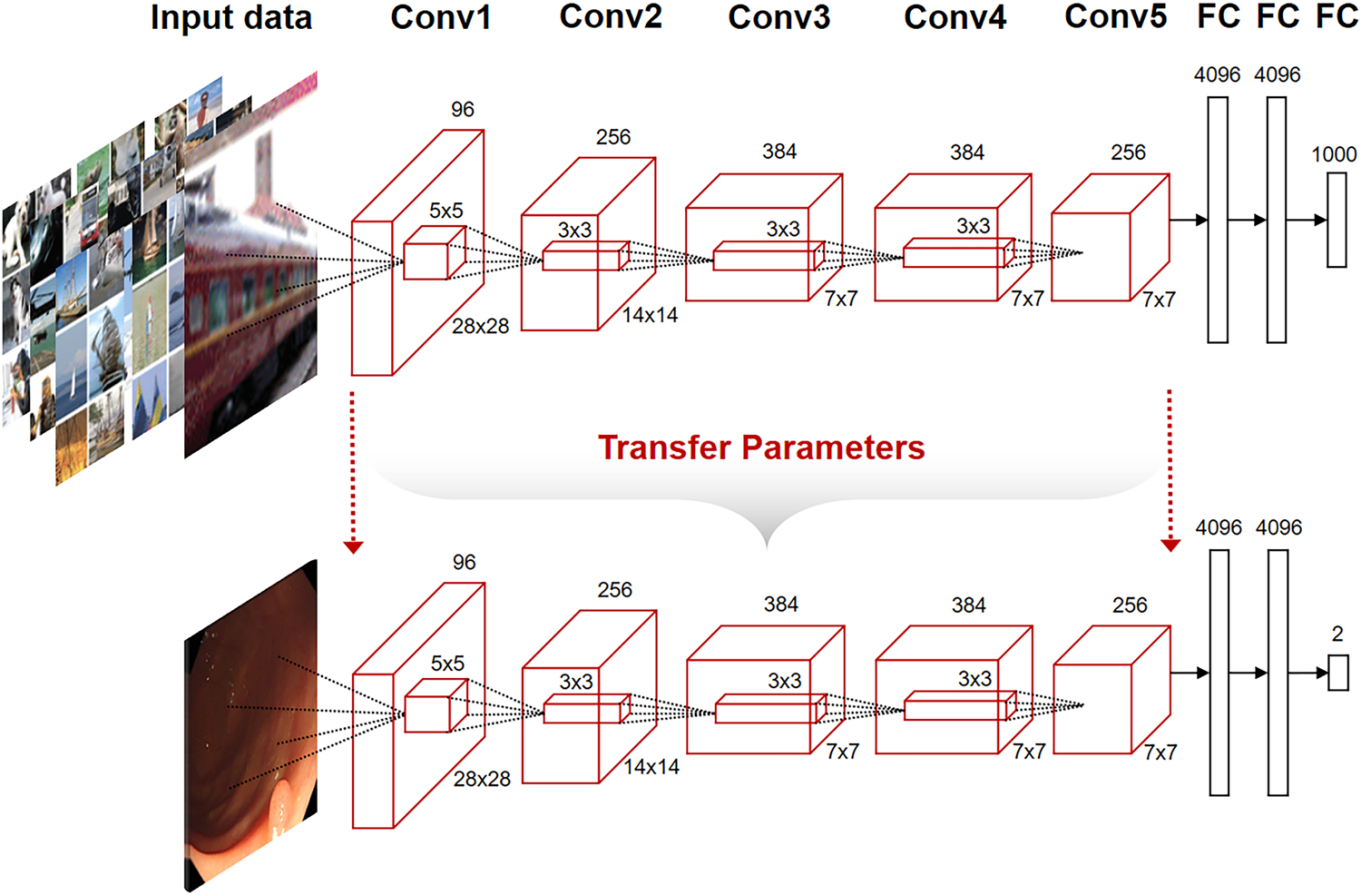
AlexNet structure of transfer learning from ImageNet database. The parameters are transferred in all layers from Conv1 to Conv5 except FC.

One wishes to learn the representation either in a purely unsupervised way or by using labels for other tasks, since labels for the task of interest are not available at the time of learning the representation. This type of setup has been called self-taught learning^20^ but also falls in the area of transfer learning. In deep learning, the aim of transfer learning is to use more abstract features in the higher levels of the representation, which hopefully make it easier to separate the various explanatory factors extent in the data^21^. In the proposed method, since the number and variety of endoscopic images with labels is limited, the already constructed deep-learning structure is adapted as a pre-trained model. The constructed structure for the pre-trained model may be appropriate as an endoscopic image classifier. Transfer learning in AlexNet transfers structures and parameters in all convolutional layers except for the fully connected (FC) layer of models pre-trained with databases. We retrained a new model for polyp classification from the transferred structures and parameters (Fig. 2).

We use the deep features from the response of fully connected (FC) layer 7 of the CNNs, which is the final fully connected layer before the class predictions are produced. There is only a minor difference between the features of FC7 and the features of the FC6 layer^17^. The deep feature for each image is a 4096-dimensional vector, if an adaptation layer is added between the FC7 layer and FC8 layer. This addition of one layer was used in other transfer-learning research studies^22^. The effect of this addition was also experimented on using Places205 and SOS datasets.

An implementation of the Salient Object Subtilizing (SOS) method using an end-to-end CNN classifier attained 94% accuracy in detecting the existence of salient objects^18^. The polyp problem can be considered as the classification of polyp existence, and this problem has aspects similar to the salient object existence problem. However, in polyp classification problems, the shape of the polyp is more restricted than an arbitrary object.

### Network-in-network-based transfer learning

Owing to the typically small spatial support for max pooling, the spatial invariance is only realized over a deep hierarchy of max pooling and convolutions, and the intermediate feature maps (convolutional layer activations) in a CNN are not actually invariant to large transformations of the input data. This limitation of CNNs is owing to having only a limited, predefined pooling mechanism for dealing with variations in the spatial arrangement of data^23^. The convolution filter in a CNN is a Generalized Linear Model (GLM) for the underlying data patch, and we argue that the level of abstraction is low in a GLM. By abstraction, we mean that the feature is invariant to the variants of the same concept^24^. In Network In Network, the GLM is replaced with a micro network structure, which is a general nonlinear function approximator. The execution of a micro network is as follows:

1 $${f}_{i,j,{k}_{1}}^{1}=max\left({W}_{{k}_{1}}^{1 T}{X}_{i,j}+ {b}_{{k}_{1}}, 0\right) \cdots {f}_{i,j,{k}_{n}}^{n}=max\left({W}_{{k}_{n}}^{n T}{f}_{i,j}^{n-1}+ {b}_{{k}_{n}}, 0\right)$$

Here, n is the number of layers in the multilayer perceptron. The resulting structure for a micro network is called an mlpconv layer. The mlpconv maps the input local patch to the output feature vector with a multilayer perceptron (MLP) consisting of multiple fully connected layers with nonlinear activation functions^15^. The used structure of the NIN is a stacking of multiple mlpconv layers. Therefore, this structure can preserve the spatial invariance more strongly than a CNN.

Instead of adding fully connected layers on top of the feature maps, NIN takes the average of each feature map, and the resulting vector is fed directly into the softmax layer. Global average pooling sums the spatial information; thus, it is more robust to spatial translations of the input. Since the location of a polyp in a recorded video of a colonoscopy is not fixed, the classification of a polyp needs considerable spatial invariance compared with another experiment that was performed under a fixed image architecture^10^.

## Results

### Polyp classification results

Table 1 lists the evaluation results of five algorithms from 20 experiments made by the random shuffling of 1000 images. AlexNet classification was performed without application of a pre-trained model for transfer learning, and AlexNet’s weights were initialized from random noise. This model produced the lowest accuracy compared with other models. After applying a pre-trained model from three public datasets (ImageNet, Places205, SOS) to AlexNet, evaluations were performed. AlexNet transfer learning to the SOS dataset (AlexNet + SOS) produced the best accuracy. AlexNet transfer learning to the Places205 dataset (AlexNet + Places) produced the worst accuracy compared with AlexNet + SOS and AlexNet transfer learning to the ImageNet dataset (AlexNet + ImageNet). The Places205 database was not appropriate for polyp classification, while Zhang et al.’s research produced nice results by transferring PlaceNet’s convolution layer weights for the support vector machine classifier^10^.

**Table 1:** Detection result of five algorithms.

	Precision	Recall	f1	TPR	FPR	Accuracy
AlexNet (A_1_)	0.627 ± 0.106	0.839 ± 0.196	0.689 ± 0.074	0.839 ± 0.196	0.571 ± 0.292	0.634 ± 0.076
AlexNet + Places (A_2_)	0.628 ± 0.043	0.950 ± 0.026	0.755 ± 0.027	0.950 ± 0.026	0.570 ± 0.116	0.690 ± 0.050
AlexNet + SOS (A_3_)	0.737 ± 0.061	0.893 ± 0.052	0.804 ± 0.027	0.893 ± 0.052	0.330 ± 0.102	0.782 ± 0.037
AlexNet + ImageNet (A_4_)	0.748 ± 0.081	0.867 ± 0.155	0.786 ± 0.097	0.867 ± 0.155	0.318 ± 0.131	0.775 ± 0.056
NIN + ImageNet (A_5_)	0.776 ± 0.080	0.929 ± 0.029	0.842 ± 0.041	0.929 ± 0.029	0.283 ± 0.127	0.823 ± 0.055

Number of iteration is 10,000.

**Figure 3:**
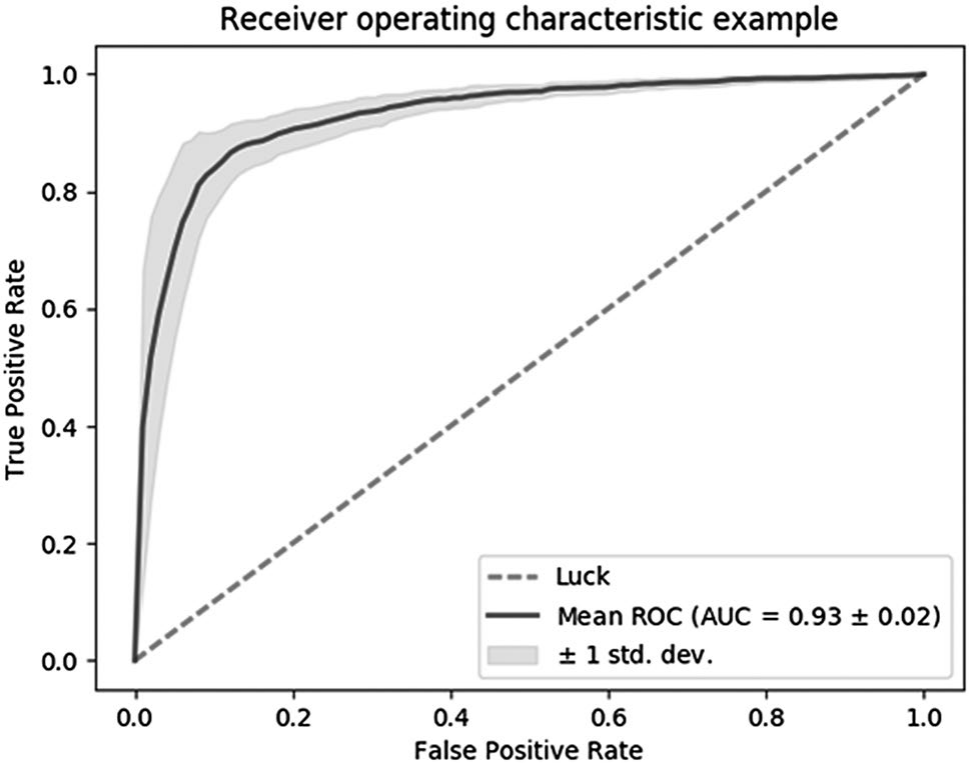
ROC curve of NIN + ImageNet model.

NIN transfer learning to the ImageNet dataset (NIN + ImageNet) used a graph structure, which had better spatial invariance, and adapted ImageNet as a pre-trained model such as AlexNet + ImageNet. NIN + ImageNet showed the best accuracy (0.823 ± 0.055) compared with the other algorithms, and gave an average area under curve (AUC) of 0.930 ± 0.020 (Fig. 3). The NIN + ImageNet system produced a high recall rate (0.929 ± 0.029) compared with the precision value (0.776 ± 0.080). By applying the advantage of a high recall rate, a radiologist can remove false positives after the first automatic processing of polyp classification, and a high recall rate can improve the usefulness of the system by reducing the number of missed polyps. Tajbakhsh et al.’s research showed a low recall rate (< 0.75)^12^, although this was applied to an entire video sequence, and Park et al.’s research gave a recall rate of 0.828 and precision of 0.658^11^. By comparison, our system’s superiority can be found from its high recall rate.

**Figure 4:**
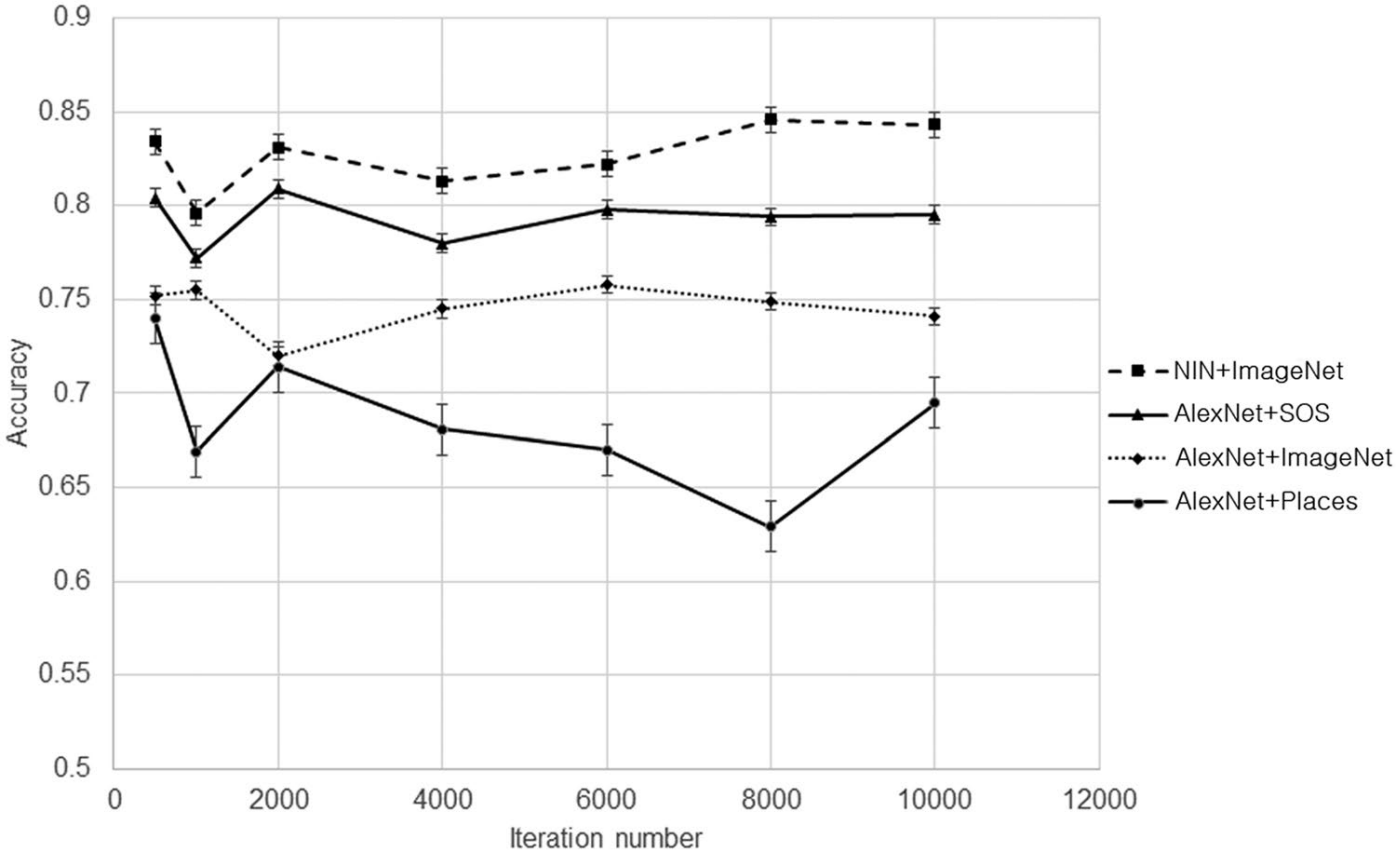
Accuracy trajectory of five systems according to changes in iteration number. Ranges for standard deviation are indicated by bars in NIN + ImageNet, AlexNet + SOS, and AlexNet + ImageNet.

A two-tail paired t-test was performed between NIN + ImageNet and other algorithms in an accuracy index. NIN + ImageNet performed significantly better than AlexNet (p < 0.001), AlexNet + Places (p < 0.001), AlexNet + ImageNet (p < 0.05), and AlexNet + SOS (p < 0.01). The P values were expressed by thresholds of 0.001, 0.01, and 0.05. The accuracy trajectory according to changes in the iteration number of training is shown in Fig. 4. By using a pre-trained model for transfer learning, the starting iteration number under the convergence of training is low, and this convergence comes before the 500th iteration. Since the system adjusts only the weights of fully connected layers by performing training with polyp images, and reuses weights on other layers from the pre-trained models, this convergence comes early. In checking the training curve of the Caffe system, all 20 cases of AlexNet converged near 2000 iterations. Although we can improve upon the low accuracy of AlexNet by changing system parameters and activation functions, this system is directly proposed for comparison with transfer-learning-based systems.

The accuracy of AlexNet + Places did not improve (0.673 ± 0.071) if an adaptation layer was added between the FC7 layer and FC8 layer. However, the accuracy of AlexNet + SOS increased when one layer was added (“Add of FC9” row of Table 2). A slight increase did not change the accuracy order of the three databases. In the AlexNet based method, the effect from the inclusion or exclusion of transferring weights in each layer of fully connected layers is presented in Table 2.

**Table 2:** AlexNet + SOS test results according to inclusion or exclusion of fully connected layers in weight transfer.

	Precision	Recall	f1	TPR	FPR	Accuracy
No transfer of fc6, fc7	0.685 ± 0.077	0.807 ± 0.140	0.729 ± 0.052	0.807 ± 0.140	0.396 ± 0.145	0.706 ± 0.041
Transfer of fc6	0.770 ± 0.101	0.793 ± 0.139	0.766 ± 0.061	0.793 ± 0.139	0.272 ± 0.186	0.761 ± 0.062
Transfer of fc6 and fc7	0.737 ± 0.061	0.893 ± 0.052	0.804 ± 0.027	0.893 ± 0.052	0.330 ± 0.102	0.782 ± 0.037
Addition of fc9	0.766 ± 0.066	0.868 ± 0.069	0.809 ± 0.034	0.868 ± 0.069	0.278 ± 0.117	0.795 ± 0.045

### Analysis of problem characteristics in polyp classification

Since 20 experiments made from random shuffling are applied to different algorithms with the same division of testing and training, the conditional probability between algorithms can be calculated by counting the errors of algorithms and true labels in each case. By analyzing the conditional probability between several algorithms, we can determine the dependence of error cases between algorithms, and the existence of difficult subsets is assumed to show the difficulty of determining polyp classification with regard to spatial variation.

The ratio in Table 3 shows the conditional probability P(A_col_ is wrong|A_row_ is wrong). A_col_ represents an algorithm under the column. A_row_ represents an algorithm under the row. By counting error cases that show wrong results on A_3_, A_4_, and A_5_, we can calculate P(A_3_,A_4_ is wrong|A_5_ is wrong). From experiments, P(A_3_,A_4_ is wrong|A_5_ is wrong) is 38.98%, and P(A_2_,A_3_,A_4_ is wrong|A_5_ is wrong) is 32.77%. Additionally, P(A_1,_A_2_,A_3_,A_4_ is wrong|A_5_ is wrong) is 23.87%. If P(A_1_ |A_5_), P(A_2_|A_5_), P(A_3_|A_5_), and P(A_4_|A_5_) are independent, the following equation is satisfied:

2 $$ {\text{P}}\left( {{\text{A}}_{{1}},\,{\text{A}}_{{2}},\,{\text{A}}_{{3}},\,{\text{A}}_{{4}}\,{\text{is wrong}}\left|{{\text{A}}_{{5}}\,{\text{is wrong}}} \right.} \right) \, = {\text{ P}}\left( {{\text{A}}_{{4}} |{\text{A}}_{{5}} } \right) \, *{\text{ P}}\left( {{\text{A}}_{{3}} |{\text{A}}_{{5}} } \right) \, *{\text{ P}}\left( {{\text{A}}_{{2}} |{\text{A}}_{{5}} } \right) \, *{\text{ P}}\left( {{\text{A}}_{{1}} |{\text{A}}_{{5}} } \right). $$

**Table 3:** Conditional probability P(A_col_ is wrong|A_row_ is wrong).

	NIN + ImageNet	AlexNet + ImageNet	AlexNet + SOS	AlexNet + Places	AlexNet
NIN + ImgaeNet	1	0.516	0.544	0.694	0.605
AlexNet + ImageNet		1	0.615	0.670	0.557
AlexNet + SOS			1	0.692	0.553
AlexNet + Places				1	0.659
AlexNet					1

Lower triangle contents of this table are ignored because of inclusion relationship of errors between algorithms.

Under an independent assumption, P(A_3_, A_4_ is wrong|A_5_ is wrong) is 28.07% , and P(A_2_,A_3_,A_4_ is wrong|A_5_ is wrong) is 19.48%. P(A_1_,A_2_,A_3_,A_4_ is wrong|A_5_ is wrong) is 11.79% from Table 3. The Joint Conditional Probability (JCP) under an independent assumption shows a comparison point. The small ratio from the division of independent JCP by JCP means that there are dependent cases in classification. There was a difference of (100–72.01% =) 27.99% in P(A_3_,A_4_ is wrong | A_5_ is wrong) from an independence assumption. We assume that there is a dependence of approximately 30% between A_3_, A_4_, and A_5_. When the characteristics of polyp classification are considered, these dependent cases can be considered as cases with large spatial variances.

## Discussion and conclusion

Recently, artificial intelligence (AI) has been used in many fields, and the medical field is no exception. Until now, many attempts such as the analysis of big data, prediction of disease, determination of treatment policy (such as Watson for oncology), and assistive purposes for image-based diagnosis in the field of medicine.

Colonoscopy is one of the most important diagnostic methods for the screening of colorectal cancer as well as for treatment purposes. However, because colonoscopy is a subjective test method, the reliability of the test results varies and depends on the degree of proficiency or concentration of the endoscopist. Therefore, quality control of colonoscopy is an essential element for colonoscopy to be used as a population-based colon cancer prevention method.

Thus far, many attempts have been made to improve the quality of colorectal cancer (PMID: 25480100, 25448873, 22987217). The adenoma detection rate (ADR) is a very important endoscopic quality indicator, and various quality indicators such as appropriate intestinal cleansing and examination time are also important to maintain an appropriate level of ADR. From this point of view, AI-assisted colonoscopy could be an alternative method.

The reason for the low performance of AlexNet + Places can be found in the characteristics of the pre-trained model database in transfer learning. AlexNet + Places produced the highest recall rate, but also had a large false positive rate since AlexNet + Places diagnosed polyps in the most cases. This bias is assumed to come from the characteristics in which the Places data deals with scene-centric data. Zhou et al. introduced a new benchmark with millions of labeled images, the Places database, designed to represent places and scenes found in the real world^17^. Categories of this database are constructed by comparing the similarity between images. This demonstrates that object-centric and scene-centric neural networks differ in their internal representations, by introducing a simple visualization of the receptive fields of CNN units.

At ILSVRC 2012, Alex et al. won the image classification test with a top-five classification error of 16.4%^16^. The evaluator checks if the target label is one of the top five predictions for the top-five classification error. For an untrained annotator, it is an extremely challenging task to annotate images from one of 1000 categories. The most common error to which an untrained annotator is susceptible is the failure to consider a relevant class as a possible label because the annotator is unaware of its existence^25^. Therefore, a top-five classification error is generated if the result class is not included in the five with the highest probability. AlexNet + ImageNet produced a 22.5% error, which is lower than the top-five classification error among 1000 classes. The classification error is assumed to be lower than the top-five error of ILSVRC since the polyp scale can vary when compared with the entire image. Additionally, the classification needs special medical knowledge.

The SOS database contains COCO^25^, ImageNet^16^, VOC07^26^, and a scene dataset, SUN^26^. The SUN dataset does not contain obviously salient objects, as it is composed of scene images. A classification network based on the SOS database finds the number of salient objects in images by annotating an image by 0 to 4+ . The object number distributions of the images from COCO and VOC07 are very similar, and the majority of images from the SUN dataset belong to the “0” category^18^. The ImageNet dataset contains more images with three salient objects than the other datasets. The average precision in the SOS problem was 0.69, and this value is lower than the precision (0.737 ± 0.061) of AlexNet + SOS. This comparison comes from a condition that finding the number of salient objects is difficult work. The recognition accuracy in the presence of salient objects was 86.5%, and this value is larger than AlexNet + SOS’s accuracy (0.782 ± 0.037). This means that the polyp recognition is more difficult than the recognition of salient objects in images.

This research improved the performance of polyp classification through the fine-tuning of Network-in-Network (NIN) after applying a pre-trained model of the ImageNet database. Three compared methods were constructed from AlexNet by transferring the weights trained by three different state-of-the-art databases. The proposed method showed a significant improvement in performance compared with the other methods.

However, AlexNet is one of the old deep learning structures in the rapidly changing field of deep learning. Therefore, we will further verify the proposed model by comparing it with the latest models such as ResNet and DenseNet in future studies. In addition, this paper focused on classification performance in colonoscopy images. However, for the practical use of the proposed method in colonoscopy, real-time processing should be possible, which should be treated not only the performance of the deep learning model but also the speed. Therefore, we will verify the processing performance of the proposed model in the future and conduct further research on real-time processing. In addition, further research on preprocessing technology based on image processing technology is needed to solve problems such as noise that may occur in images. In order to utilize artificial intelligence in colonoscopy, it is necessary to automatically recognize polyps, to distinguish the types of polyps, and to provide a diagnosis of benign and malignant to a clinician. Therefore, further studies on models that can diagnose malignancy and benignity based on various types of differentiating polyps should be conducted, and verification of generalization should be performed using various public data.

In order to utilize artificial intelligence in colonoscopy, many processes are required. The proposed method is to recognize polyps, which is only the initial stage in light of the whole process, but it can confirm the sufficient possibility in its performance. In the future, if further studies are conducted to diagnose malignancy and benignity according to real-time treatment and types of polyps, artificial intelligence technology is expected to be helpful for colonoscopy.
